# Ingesting Yogurt Containing *Lactobacillus plantarum* OLL2712 Reduces Abdominal Fat Accumulation and Chronic Inflammation in Overweight Adults in a Randomized Placebo-Controlled Trial

**DOI:** 10.1093/cdn/nzab006

**Published:** 2021-02-03

**Authors:** Takayuki Toshimitsu, Ayako Gotou, Toshihiro Sashihara, Keisuke Furuichi, Satoshi Hachimura, Nobuhiko Shioya, Satoru Suzuki, Yukio Asami

**Affiliations:** Applied Microbiology Research Department, Food Microbiology Research Laboratories, Division of Research and Development, Meiji Co., Ltd., Hachiouji, Tokyo, Japan; Applied Microbiology Research Department, Food Microbiology Research Laboratories, Division of Research and Development, Meiji Co., Ltd., Hachiouji, Tokyo, Japan; Applied Microbiology Research Department, Food Microbiology Research Laboratories, Division of Research and Development, Meiji Co., Ltd., Hachiouji, Tokyo, Japan; Applied Microbiology Research Department, Food Microbiology Research Laboratories, Division of Research and Development, Meiji Co., Ltd., Hachiouji, Tokyo, Japan; Research Center for Food Safety, Graduate School of Agricultural and Life Sciences, The University of Tokyo, Bunkyo-ku, Tokyo, Japan; Statistical Analysis Department, KSO Corporation, Minato-ku, Tokyo, Japan; Shinagawa Season Terrace Health Care Clinic, Minato-ku, Tokyo, Japan; Applied Microbiology Research Department, Food Microbiology Research Laboratories, Division of Research and Development, Meiji Co., Ltd., Hachiouji, Tokyo, Japan

**Keywords:** abdominal fat area, chronic inflammation, fasting plasma glucose, high-sensitivity C-reactive protein, HOMA-IR, interleukin-6, insulin resistance, *Lactobacillus plantarum*, yogurt

## Abstract

**Background:**

Chronic inflammation and insulin resistance are factors that are related to obesity. We have suggested that the administration of heat-treated *Lactobacillus plantarum* OLL2712 (OLL2712) cells can improve glucose and lipid metabolism by suppressing chronic inflammation in mouse models and a preliminary clinical study.

**Objective:**

The aim of this study was to investigate whether ingesting OLL2712 cells can reduce body fat accumulation and improve metabolic risk factors, in overweight, healthy adults.

**Methods:**

This study was a randomized, double-blind, placebo-controlled, parallel-group trial conducted at a single center in Japan. The study participants included 100 overweight (BMI range, ≥25 to <30 kg/m^2^) adults aged 20–64 y. They were randomly assigned to either the placebo or OLL2712 group (*n* = 50 each) and were administered conventional yogurt or yogurt containing >5 × 10^9^ heat-treated OLL2712 cells, respectively, daily for 12 wk. The primary outcome was the 12-wk change in the abdominal fat area, as assessed by computed tomography, and the secondary outcomes were glucose and lipid metabolism-related parameters and chronic inflammation markers, which were analyzed using a linear mixed model.

**Results:**

The 12-wk change of abdominal fat area (difference: 8.5 cm^2^; 95% CI: 0.3, 16.6 cm^2^; *P *= 0.040) and fasting plasma glucose (difference: 3.2 mg/dL; 95% CI: 0.8, 5.6 mg/dL; *P *= 0.021) were significantly less in the OLL2712 group than the placebo group. The overall trend of serum IL-6 was significantly decreased in the OLL2712 group compared with baseline and the placebo group.

**Conclusions:**

The ingestion of heat-treated OLL2712 cells reduces body fat accumulation and the deterioration of glycemic control and chronic inflammation, in overweight, healthy adults. We hypothesize that OLL2712 cells may prevent obesity by regulating chronic inflammation. This trial was registered at the University Hospital Medical Information Network Clinical Trials Registry as UMIN000027709.

## Introduction

Currently, ∼15% of the world's population is obese and 5–10% have type-2 diabetes mellitus (T2DM), and these rates are continually increasing (1, 2). Obesity causes lifestyle-related metabolic disorders, including hypertension, dyslipidemia, and T2DM (3). Chronic low-grade inflammation and insulin resistance (IR) are closely associated with the development of obesity and metabolic disorders (4). An increased production of proinflammatory cytokines has been reported to induce chronic inflammation in the adipose, muscle, and liver tissues, which can cause IR as well as impaired glucose and lipid metabolism (5). Therefore, the alleviation of chronic inflammation via the suppression of proinflammatory cytokines could improve lipid metabolism and reduce the accumulation of body fat.

Improvements in dietary and exercise habits can be effective in reducing the risk of obesity. However, as intensive lifestyle interventions are difficult to maintain over the long term, the development of effective, low-cost, lifestyle interventions that facilitate high adherence rates are needed. The antiobesity effects of functional foods have been attracting public attention (6). Previous studies have shown that certain lactic acid bacteria (LAB) strains improve lipid metabolism and reduce body fat in both animal studies and clinical trials (7, 8). The mechanisms by which LAB reduce body fat have not yet been fully determined in previous clinical studies; however, the suppression of chronic inflammation is one of the presumed mechanisms that functions against obesity and metabolic disorders (9, 10). Thus far, no previous clinical studies have demonstrated that certain LAB preclude the accumulation of body fat via the suppression of chronic inflammation.

Several clinical trials have reported that the ingestion of live LAB cells suppresses body fat accumulation in overweight individuals (11, 12). These studies have upheld that the mechanisms underpinning body fat reduction were related to improvements in intestinal bacterial flora, greater production of SCFAs (11), and suppression of lipid absorption in the intestinal tract (12). On the other hand, it has been reported that body fat can be reduced by the ingestion of heat-treated bacteria (13). It was reported that the administration of heat-treated *Akkermansia muciniphila* cells reduced chronic inflammation, IR, and body fat accumulation more effectively than live bacterial cells (14). They revealed that the effective molecule in *A. muciniphila* is a cell-surface membrane protein that stimulates the toll-like receptor 2 (TLR2)-mediated activation of immune cells and showed that heat treatment enhances the anti-inflammatory activity of the bacteria (15, 16). So far, no clinical trials have clarified the antiobesity effects of heat-treated LAB.

In our previous study, we selected *Lactobacillus plantarum* OLL2712 (OLL2712) for its strong ability to induce IL-10 production in the intestinal immune system and to suppress chronic inflammation in the adipose tissue (17, 18). The IL-10-inducing activity of heat-treated OLL2712 cells was higher than that of live cells and was TLR2 activity dependent (19). IL-10 suppresses the production of proinflammatory cytokines (20, 21). Accordingly, the induction of IL-10 in the intestine may prevent chronic inflammation in the adipose tissue (22, 23). The administration of heat-treated OLL2712 cells also alleviated chronic inflammation and improved hyperlipidemia and hyperglycemia in murine models of obesity and T2DM (19). In a preliminary clinical study, we demonstrated that heat-treated OLL2712 cells reduced fasting plasma glucose (FPG), body fat, and serum proinflammatory cytokine concentrations compared with those at baseline in prediabetic individuals (24).

We hypothesized that heat-treated OLL2712 cells would prevent obesity by suppressing chronic inflammation and IR. In the present study, we investigated whether long-term ingestion of yogurt containing heat-treated OLL2712 cells could reduce body fat accumulation and improve metabolic risk factors, including chronic inflammation markers, in overweight healthy adults with a BMI of 25 to <30 kg/m^2^.

Globally, yogurt is a relatively affordable dairy food containing various nutrients (25) and can be easily ingested as a daily meal. Additionally, yogurt has been reported to be effective for reducing the risk of obesity and other metabolic disorders (26–28), which supports our rationale for selecting yogurt as a suitable test food for this study.

## Methods

### Study design

This was a randomized, double-blind, placebo-controlled, parallel-group design study conducted at a single site in Minato-ku, Tokyo, Japan, between July and December 2017. The data were analyzed in March 2018. This study was conducted in accordance with the Declaration of Helsinki, and the investigation protocol was approved by the Ethical Committee of Shinagawa Season Terrace Health Care Clinic and the Meiji Institutional Review Board. Written informed consent was obtained from each participant prior to screening. The study protocol was enrolled in the University Hospital Medical Information Network (UMIN) Clinical Trials Registry (UMIN000027709) on 12 June, 2017. The reporting of this trial follows recommendations of the Consolidated Standards of Reporting Trials 2010 statement (29).

### Participants

Study participants were recruited from a volunteer database associated with a contract research organization. All participants were Japanese and living in the Tokyo metropolitan area. For screening criteria, eligible participants had to be overweight (BMI range, ≥25 to <30 kg/m^2^) but otherwise healthy adults aged between 20 and 64 y. Candidates were excluded if they had: *1*) an obesity-related disease, *2*) a food allergy or lactose intolerance, *3*) habitually consumed fermented milk or a lactic fermenting beverage 3 or more times weekly within 3 mo before screening, *4*) habitually ingested oral medication, supplements, or health foods affecting obesity, hyperlipidemia, glucose, or lipid metabolism within 3 mo before screening, *5*) a regular regimen of performing intense exercise, *6*) drug or alcohol dependency, *7*) an excessive alcohol intake or were unable to abstain from drinking 2 d before screening, *8*) metal implants at the computed tomography (CT) scan site, *9*) a cardiac pacemaker and/or implantable defibrillator, *10*) familial hyperlipidemia, *11*) became pregnant or were breastfeeding, *12*) extremely irregular eating habits, *13*) participated in other clinical trials within 1 mo before agreeing to participate in this study, *14*) claustrophobia, or *15*) were judged as unsuitable for the study by the principal investigator for other reasons.

The target number of participants for enrollment was 100 (i.e. 50 participants per group), which enabled us to perceive a group difference in the change of body fat percentage at an α = 0.05 level and a power of 80%. The sample size calculation was performed based on the body fat percentages of prediabetic participants in a preliminary clinical study (24); the participants whose BMI were ≥25 reduced their body fat percentages by a mean of 2.5 and SD of 4.4 (*n* = 15) after ingesting heat-treated OLL2712 cells for 12 wk. As some candidates would likely decline to participate in the study, and comorbidities or ill-health would make others ineligible to participate, we aimed to screen 352 adults.

### Randomization and blinding

Study participants were randomly allocated 1:1 to the OLL2712 or placebo group. A designated individual who was not involved in the study design, enrollment, evaluation, intervention, or analysis created a random number sequence using a generally authorized computer program (JMP; SAS Institute Japan). The participants were randomly assigned to 1 of 4 blocks based on information obtained during screening, balancing the parameters of gender, age, abdominal fat area, and BMI. This was a triple-blind study with the group allocations being withheld from participants, investigators, and statisticians until data analysis was completed.

### Test food

The test foods were 112 g of placebo yogurt or yogurt containing heat-treated OLL2712 (>5 × 10^9^ cells/112 g of yogurt). This dose was set by extrapolating the effective dose in animal studies (19) to humans. The test yogurt was manufactured at a pilot plant in Meiji Co., Ltd. on a weekly basis, delivered under refrigeration conditions to the homes of the participants, and refrigerated until consumption. The OLL2712 yogurt was confirmed to be identical in terms of appearance and flavor to the placebo yogurt. Both yogurts consisted of 58% dairy products, 3.4% sugar, 0.0038% sucralose, and a 3% culture of yogurt starters. Each yogurt provided 76 kcal, 4.5 g of protein, 1.6 g of fat, 10.8 g of carbohydrates, 0.14 g of sodium, and 0.12 g of calcium. We used heat-treated OLL2712 cells as they showed higher IL-10-inducing activity than live cells in in vitro assays using immune cells (24). The heat-treated OLL2712 cells were prepared as previously described (30).

### Interventions

The study period consisted of a 4-wk screening followed by a 12-wk treatment period. Each participant took medical interviews by the lead physician and had blood drawn 5 times in total, during screening and at 0 (baseline), 4, 8, and 12 wk. Abdominal fat areas were assessed 4 times using CT during screening and at 0, 8, and 12 wk.

Participants were instructed to take the 112-g test yogurt daily for 12 wk (from the 0- to 12-wk visits) and visit the clinic every 4 wk. During the study period, participants were asked to maintain their normal dietary and lifestyle habits, including the quality and quantity of exercise. Participants were provided with a life diary to check for compliance and to report any health issues. Participants were also asked to mention the types and amount of exercise they performed and step counts measured by a pedometer (Manpo MK-365; Yamasa) daily in their life diaries. The lead physician monitored the participants’ compliance based on interviews and the diary entries at each visit.

### Measurements

All study outcomes, except for abdominal fat area, were assessed at baseline and at each 4-wk visit thereafter. The primary outcome measures were change in the abdominal fat areas by 12 wk. The secondary outcomes were changes in body weight, BMI, body fat percentage, waist-to-hip circumference, and metabolic risk factors such as glycemic control, lipid profiles, and chronic inflammation markers. To monitor adherence to the dietary intervention, food intake was measured every 4 wk.

Abdominal fat areas were analyzed at the level of the L4 vertebrae using CT (Supria; Hitachi) and computed by a commercial program (Fat Scan ver. 5.0; e-JAPAN IT) (31). Body weight (to the nearest 0.1 kg) and body fat percentage (to the nearest 0.1%) were measured using a multifrequency bioelectrical impedance device (InBody 430; Biospace). Height was measured to the nearest 0.1 cm by utilizing a compact stadiometer (DSN-90; Muratec-KDS). BMI was estimated from participants’ weight (kg)/height (m^2^). Waist and hip circumference were measured twice while in a standing position to the nearest 0.1 cm at the umbilicus and maximum circumference of the buttocks, respectively, using a flexible plastic tape. The average value of the 2 measurements was used for analysis. The waist-to-hip ratio was calculated from participants’ waist circumference divided by their hip circumference.

Following an overnight fasting (≥10 h), a blood sample was drawn from the antecubital vein of each participant. Venous blood was assayed by an independent laboratory (LSI Medience). The serum total cholesterol, serum LDL cholesterol, serum HDL cholesterol, serum triglycerides, plasma glucose, serum insulin, and whole blood glycated hemoglobin (HbA1c) were measured as previously described (30). Serum ketone bodies were measured using an enzyme cycling method (KAINOS TKB-L; Kainos Laboratories). Serum high-sensitivity C-reactive protein (hs-CRP) was measured using the nephelometry method (N-latex CRPⅡ; Siemens Healthineers Japan). The HOMA-IR was calculated according to the following formula: HOMA-IR = fasting glucose (mg/dL) × fasting insulin (μU/mL)/405 (32).

Serum proinflammatory cytokines were measured using a multiplex human cytokine bead array system (Bio-Rad). Serum adiponectin was measured using the adiponectin enzyme-linked immunosorbent assay kit (Alpco). Systolic and diastolic blood pressure readings were measured as previously described (30).

### Diet records

Participants recorded their diet in questionnaires according to detailed written and verbal instructions so that their macronutrient intake could be calculated for 3 d; this included 2 weekdays and 1 weekend day before each clinical examination. Food quantities were measured using standard measuring glasses, spoons, and digital scales. These records were used to assess habitual energy intake and diet composition. Diet records were analyzed using Excel Eiyokun ver. 8.0 (Kenpakusha).

### Safety assessment

At each visit, the lead physician assessed the participant's health status through a medical interview and the diary entries. Hematology, blood biochemical, and urinalysis tests were performed at the 0- and 12-wk visits. Adverse events were reported by participants in diary entries or obtained via laboratory blood test results. The relatedness of the adverse events to the study and their seriousness were judged by the physician.

### Statistical analyses

All analyses were performed using SAS ver. 9.4 (SAS Institute Japan). *P* values < 0.05 were considered statistically significant. The data are presented as means ± SD or SE. The group-by-time interaction and intergroup differences were evaluated using a linear mixed model with the model using the amount of change (not including 0 wk) as the response variable, group, time, and group-by-time interaction as fixed effects, the baseline value as the covariate, and time point as a repeated effect. The variance-covariance matrix of unstructured form (UN) was used in order to model the correlation, within each participant, between repeated measurements. Outcomes made at 12 wk were considered a priori to be primary with 4- and 8-wk measures considered as secondary reference measures and, therefore, comparisons at 12 wk were made regardless of interim results. Only when the group-by-time interaction was significant, the tests at 4 and 8 wk were performed. The intergroup comparisons at 4, 8, and 12 wk were performed using post hoc tests associated with the linear mixed model. The Shapiro–Wilk test for normality was performed, and as appropriate, the paired samples *t*-tests or Wilcoxon signed-rank tests were used to compare intragroup changes. The incidence of adverse events between groups was analyzed using Fisher's exact test.

## Results

### Participant characteristics

**FIGURE 1:**
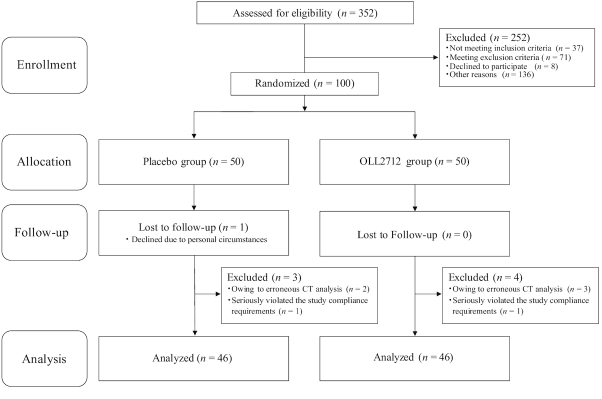
Flow chart of study participants. The placebo group received conventional yogurt and the OLL2712 group received yogurt containing >5 × 10^9^ heat-treated *Lactobacillus plantarum* OLL2712 cells. CT, computed tomography.

A flow chart of the study participants is depicted in **Figure 1**. Altogether, 352 candidates were screened and provided written informed consent for study participation. Of these, 108 were ineligible for study inclusion after not meeting the inclusion criteria or due to the exclusion criteria, 8 declined to participate, and 136 were excluded for other reasons. A total of 100 individuals without any disease or a diagnosis of obesity by the principal investigator were selected in order based on proximity to an abdominal visceral fat area of 100 cm^2^ and were assigned randomly to the OLL2712 group (*n* = 50) or the placebo group (*n *= 50). Of the 100 participants, 99 completed the 12-wk intervention period and among them, the rate of adherence to the test yogurts was similar between groups (>95%). After the exclusion of 7 participants (*n* = 5 owing to erroneous CT analysis and *n* = 2 seriously violated the study compliance requirements), 92 participants were included in the per-protocol set (PPS) analysis. When analyzed by intent-to-treat (ITT), the data of inappropriate participants judged by the principal investigator and erroneous CT analysis would also be included, resulting in an incorrect interpretation. Therefore, we believe that PPS analysis is appropriate for this study. The characteristics of the participants measured at baseline (0 wk) are presented in **Table 1**.

**TABLE 1:** Baseline characteristics of study participants (PPS analysis)^1^

	Placebo (*n* = 46)	OLL2712 (*n* = 46)
Anthropometrics		
Age, y	44.7 ± 8.2	45.5 ± 10.7
Gender, male/female, *n*	33/13	30/16
Weight, kg	77.3 ± 8.9	74.4 ± 8.2
Height, cm	168 ± 9	165 ± 8
BMI, kg/m^2^	27.5 ± 1.4	27.4 ± 1.3
Body fat, %	32.1 ± 6.1	32.1 ± 5.4
Waist circumference, cm	94.3 ± 4.7	93.3 ± 4.1
Hip circumference, cm	100.2 ± 3.6	99.2 ± 3.9
Waist-to-hip ratio	0.942 ± 0.04	0.941 ± 0.04
Abdominal fat area, cm^2^		
Total	337 ± 60	325 ± 56
Visceral	107 ± 21	102 ± 16
Subcutaneous	230 ± 55	223 ± 57
Glycemic control		
FPG, mg/dL	83.6 ± 6.6	86.3 ± 6.4
Whole blood HbA1c, %	5.45 ± 0.31	5.48 ± 023
Fasting serum insulin, mU/L	6.83 ± 2.38	7.31 ± 3.02
HOMA-IR	1.41 ± 0.51	1.57 ± 0.71
Serum ketone bodies, µmol/L	76.6 ± 75.3	89.4 ± 88.6
Cardiovascular disease risk factors		
Systolic blood pressure, mmHg	120 ± 16	119 ± 12
Diastolic blood pressure, mmHg	71.4 ± 10.9	69.9 ± 8.4
Heart rate, bpm	71.5 ± 11.1	74.6 ± 9.6
Serum total cholesterol, mg/dL	216 ± 34	204 ± 37
Serum LDL cholesterol, mg/dL	134 ± 28	126 ± 32
Serum HDL cholesterol, mg/dL	48.3 ± 10.4	49.4 ± 9.1
Serum triglycerides, mg/dL	142 ± 171	122 ± 62
Chronic inflammation markers		
Serum IL-6, pg/mL	0.442 ± 0.857	0.870 ± 1.518
Serum IL-8, pg/mL	6.03 ± 2.43	6.37 ± 2.10
Serum MCP-1, pg/mL	25.2 ± 9.8	34.2 ± 14.3
Serum TNF-α, pg/mL	10.1 ± 4.1	11.4 ± 3.8
Serum hs-CRP, mg/dL	0.0659 ± 0.0519	0.0712 ± 0.0736
Serum adiponectin, mg/mL	4.59 ± 1.81	4.37 ± 1.63

1 Values are means ± SD or *n*, *n* = 46 in each group.

FPG, fasting plasma glucose; HbA1c, glycated hemoglobin; hs-CRP, high-sensitivity C-reactive protein; MCP-1, monocyte chemotactic protein-1; PPS, per-protocol set.

Dietary intakes and physical activity over time are shown in **Table 2**. Nutrient intakes from the test yogurts were not included in this result. No significant intergroup differences were observed in these parameters.

**TABLE 2:** Dietary intake and physical activity in overweight adults who consumed OLL2712 or placebo yogurt for 12 wk (PPS analysis)^1^^,^^2^

						Linear mixed model, *P* value^3^	
	Group	0 wk	4 wk	8 wk	12 wk	Interaction	12 wk
Total energy, kcal/d	Placebo	1938 ± 429	1955 ± 449	1941 ± 543	1949 ± 448	0.406	0.848
	OLL2712	1774 ± 419	1896 ± 407	1808 ± 378	1825 ± 436		
Protein, g/d	Placebo	70.6 ± 18.4	72.9 ± 18.5	70.6 ± 22.5	71.0 ± 20.1	0.671	0.823
	OLL2712	65.0 ± 15.9	69.7 ± 13.0	70.0 ± 18.1	68.1 ± 18.9		
Fat, g/d	Placebo	62.7 ± 18.2	65.2 ± 21.6	64.2 ± 24.6	63.6 ± 21.1	0.193	0.866
	OLL2712	61.6 ± 23.3	66.6 ± 17.8	59.6 ± 14.6	63.7 ± 19.9		
Carbohydrates, g/d	Placebo	261 ± 63	257 ± 58	259 ± 69	260 ± 56	0.394	0.557
	OLL2712	228 ± 55	242 ± 56	235 ± 58	233 ± 60		
Dietary fiber, g/d	Placebo	11.4 ± 3.3	11.1 ± 3.0	11.3 ± 3.7	11.1 ± 3.6	0.349	0.269
	OLL2712	9.7 ± 3.0	11.0 ± 3.6	10.3 ± 3.1	10.6 ± 3.7		
Steps, count/d	Placebo	7870 ± 3364	7613 ± 2879	7487 ± 2682	7628 ± 2487	0.884	0.847
	OLL2712	7282 ± 3001	7418 ± 2950	7172 ± 2965	7291 ± 3268		

1 Values are means ± SD, *n* = 46 in each group.

2 Significant differences in measurements compared with baseline (0 wk) were determined using the paired samples *t*-tests or the Wilcoxon signed-rank tests.

3 The group-by-time interaction and intergroup differences were evaluated using a linear mixed model with the model using the amount of change (not including 0 wk) as the response variable, group, time, and group-by-time interaction as fixed effects, the baseline value as the covariate, and time point as a repeated effect.

PPS, per-protocol set.

### Effects of OLL2712 yogurt on body fat accumulation and anthropometric measurements

The abdominal total and subcutaneous fat areas significantly increased from 0 to 12 wk in the placebo group but not in the OLL2712 group (**Table 3**). The 12-wk change of abdominal total fat area, as the primary outcome, was significantly less in the OLL2712 group than the placebo group, analyzed by a linear mixed model (difference: 8.5 cm^2^; 95% CI: 0.3, 16.6 cm^2^; *P *= 0.040) (Table 3). Similar but nonsignificant results were obtained for the abdominal subcutaneous fat area measurements (difference: 5.4 cm^2^; 95% CI: −0.2, 11.1 cm^2^; *P* = 0.065) (Table 3).

**TABLE 3:** Body fat and anthropometric measurements in overweight adults who consumed OLL2712 or placebo yogurt for 12 wk (PPS analysis)^1^^,^^2^

						Linear mixed model, *P* value^3^			
	Group	0 wk	4 wk	8 wk	12 wk	Interaction	4 wk	8 wk	12 wk
Abdominal total fat area, cm^2^	Placebo	337 ± 60	NA	346 ± 62	345 ± 65**	0.296	NA	NT	0.040
	OLL2712	325 ± 56	NA	329 ± 55	324 ± 53				
Abdominal visceral fat area, cm^2^	Placebo	107 ± 21	NA	112 ± 23	109 ± 26	0.771	NA	NT	0.229
	OLL2712	102 ± 16	NA	103 ± 19	101 ± 17				
Abdominal subcutaneous fat area, cm^2^	Placebo	230 ± 55	NA	235 ± 57**	236 ± 58**	0.034	NA	0.629	0.065
	OLL2712	223 ± 57	NA	226 ± 58**	223 ± 56				
Weight, kg	Placebo	77.3 ± 8.9	77.9 ± 8.8	78.0 ± 8.9	77.9 ± 8.8**	0.971	NT	NT	0.244
	OLL2712	74.4 ± 8.2	74.6 ± 8.4	74.7 ± 8.4	74.6 ± 8.3				
BMI, kg/m^2^	Placebo	27.5 ± 1.4	27.7 ± 1.4	27.8 ± 1.5	27.7 ± 1.5**	0.976	NT	NT	0.226
	OLL2712	27.4 ± 1.3	27.5 ± 1.4	27.5 ± 1.4	27.5 ± 1.4				
Body fat, %	Placebo	32.1 ± 6.1	32.1 ± 6.2	33.2 ± 6.1	33.4 ± 6.1**	0.682	NT	NT	0.505
	OLL2712	32.1 ± 5.4	32.2 ± 5.5	32.9 ± 5.9	33.1 ± 5.3**				
Waist circumference, cm	Placebo	94.3 ± 4.7	94.3 ± 4.6	93.9 ± 4.8	93.7 ± 4.8*	0.501	NT	NT	0.396
	OLL2712	93.3 ± 4.1	93.1 ± 4.5	92.9 ± 4.2	92.4 ± 4.4**				
Hip circumference, cm	Placebo	100.2 ± 3.6	100.2 ± 3.3	100.0 ± 3.4	100.1 ± 3.7	0.484	NT	NT	0.566
	OLL2712	99.2 ± 3.9	99.2 ± 4.2	98.9 ± 4.1	99.0 ± 4.3				
Waist-to-hip ratio	Placebo	0.942 ± 0.044	0.942 ± 0.041	0.939 ± 0.041	0.936 ± 0.042	0.157	NT	NT	0.466
	OLL2712	0.941 ± 0.036	0.939 ± 0.038	0.940 ± 0.035	0.934 ± 0.035*				

1 Values are means ± SD, *n* = 46 in each group.

2 Significant differences in measurements compared to baseline (0 wk) were determined using the paired samples *t*-tests or the Wilcoxon signed-rank tests (*P < 0.05, **P < 0.01).

3 The group-by-time interaction and intergroup differences were evaluated using a linear mixed model with the model using the amount of change (not including 0 wk) as the response variable, group, time, and group-by-time interaction as fixed effects, the baseline value as the covariate, and time point as a repeated effect.

NA, not assessed; NT, not tested; PPS, per-protocol set.

Body weight and BMI, as secondary outcomes, significantly increased from 0 to 12 wk in the placebo group but not in the OLL2712 group. Body fat percentages significantly increased from 0 to 12 wk in both groups; however, waist circumference was significantly reduced from 0 to 12 wk in both groups, and waist-to-hip ratio was significantly reduced from 0 to 12 wk in the OLL2712 group but not in the placebo group (Table 3). No significant intergroup differences were observed in these parameters.

### Effects of OLL2712 yogurt on glycemic control and cardiovascular disease risk factors

FPG significantly increased from 0 to 12 wk in the placebo group but not in the OLL2712 group. The 12-wk changes in FPG were significantly less in the OLL2712 group than the placebo group (difference: 3.2 mg/dL; 95% CI: 0.8, 5.6 mg/dL; *P *= 0.021) (**Table 4**).

**TABLE 4:** Parameters related to glucose metabolism in overweight adults who consumed OLL2712 or placebo yogurt for 12 wk (PPS analysis)^1^^,^^2^

						Linear mixed model, *P* value^3^	
	Group	0 wk	4 wk	8 wk	12 wk	Interaction	12 wk
FPG, mg/dL	Placebo	83.6 ± 6.6	85.0 ± 8.3	85.7 ± 9.2	85.7 ± 9.0*	0.349	0.021
	OLL2712	86.3 ± 6.4	86.1 ± 6.8	85.8 ± 7.8	85.2 ± 7.4		
Whole blood HbA1c, %	Placebo	5.45 ± 0.31	5.42 ± 0.32	5.48 ± 0.34	5.46 ± 0.36	0.415	0.494
	OLL2712	5.48 ± 0.23	5.46 ± 0.22	5.50 ± 0.23	5.47 ± 0.23		
Fasting serum insulin, µU/mL	Placebo	6.83 ± 2.38	6.49 ± 2.12	7.22 ± 2.87	7.02 ± 2.65	0.233	0.298
	OLL2712	7.31 ± 3.02	7.13 ± 3.60	6.90 ± 2.58	6.85 ± 2.94		
HOMA-IR	Placebo	1.41 ± 0.51	1.48 ± 0.83	1.53 ± 0.64	1.50 ± 0.63	0.682	0.201
	OLL2712	1.57 ± 0.71	1.54 ± 0.84	1.48 ± 0.61	1.47 ± 0.75		
Serum ketone bodies, µmol/L	Placebo	77 ± 75	76 ± 85	78 ± 72	118 ± 163	0.273	0.484
	OLL2712	89 ± 89	72 ± 78	101 ± 88	100 ± 127		

1 Data are presented as means ± SD, *n* = 46 in each group.

2 Significant differences in measurements compared with baseline (0 wk) were determined using the Wilcoxon signed-rank tests (*P < 0.05).

3 The group-by-time interaction and intergroup differences were evaluated using a linear mixed model with the model using the amount of change (not including 0 wk) as the response variable, group, time, and group-by-time interaction as fixed effects, the baseline value as the covariate, and time point as a repeated effect.

FPG, fasting plasma glucose; HbA1c, glycated hemoglobin; PPS, per-protocol set.

The changes in cardiovascular disease risk factors are shown in **Table 5**. Heart rates significantly increased from 0 to 12 wk in the placebo group but not in the OLL2712 group. Serum HDL cholesterol significantly increased from 0 to 12 wk in the OLL2712 group but not in the placebo group. No significant intergroup differences were observed in these parameters.

**TABLE 5:** Cardiovascular disease risk factors in overweight adults who consumed OLL2712 or placebo yogurt for 12 wk (PPS analysis)^1^^,^^2^

						Linear mixed model, *P* value^3^	
	Group	0 wk	4 wk	8 wk	12 wk	Interaction	12 wk
Systolic blood pressure, mmHg	Placebo	120 ± 16	122 ± 12	121 ± 13	120 ± 13	0.189	0.934
	OLL2712	119 ± 12	122 ± 9	119 ± 12	120 ± 13		
Diastolic blood pressure, mmHg	Placebo	71.4 ± 10.9	71.7 ± 11.6	73.6 ± 9.6	73.3 ± 8.9	0.332	0.814
	OLL2712	69.9 ± 8.4	70.1 ± 8.6	70.6 ± 9.0	72.2 ± 9.2		
Heart rate, bpm	Placebo	71.5 ± 11.1	74.2 ± 11.8	73.6 ± 9.8	75.9 ± 10.5**	0.713	0.241
	OLL2712	74.6 ± 9.6	74.8 ± 10.3	73.3 ± 10.8	75.5 ± 12.2		
Serum total cholesterol, mg/dL	Placebo	216 ± 34	223 ± 34	219 ± 35	215 ± 37	0.745	0.547
	OLL2712	204 ± 37	208 ± 37	206 ± 38	202 ± 36		
Serum LDL cholesterol, mg/dL	Placebo	134 ± 28	136 ± 31	135 ± 28	137 ± 33	0.495	0.402
	OLL2712	126 ± 32	125 ± 32	127 ± 34	126 ± 32		
Serum HDL cholesterol, mg/dL	Placebo	48.3 ± 10.4	50.0 ± 10.3	49.8 ± 9.9	49.9 ± 9.4	0.924	0.448
	OLL2712	49.4 ± 9.1	51.3 ± 8.0	51.2 ± 8.9	51.6 ± 9.0**		
Serum triglycerides, mg/dL	Placebo	142 ± 171	158 ± 145	157 ± 162	143 ± 157	0.838	0.273
	OLL2712	122 ± 62	135 ± 106	127 ± 75	116 ± 49		

1 Values are means ± SD, *n* = 46 in each group.

2 Significant differences in measurements compared with baseline (0 wk) were determined using the paired samples *t*-tests or the Wilcoxon signed-rank tests (^**^P < 0.01).

3 The group-by-time interaction and intergroup differences were evaluated using a linear mixed model with the model using the amount of change (not including 0 wk) as the response variable, group, time, and group-by-time interaction as fixed effects, the baseline value as the covariate, and time point as a repeated effect. PPS, per-protocol set.

### Effects of OLL2712 yogurt on chronic inflammation

Serum IL-6 significantly decreased from 0 to 4 and 12 wk in the OLL2712 group but not in the placebo group, and the group-by-time interaction was significant (**Table 6**). Serum IL-8 significantly increased in the placebo group from 0 to 12 wk but not in the OLL2712 group. Other cytokines did not change significantly from 0 to 12 wk in either group. In addition, serum hs-CRP significantly increased in the placebo group from 0 to 12 wk but not in the OLL2712 group. Serum adiponectin significantly increased in the OLL2712 group from 0 to 12 wk but not in the placebo group (Table 6).

**TABLE 6:** Serum proinflammatory cytokines, hs-CRP, and adiponectin in overweight adults who consumed OLL2712 or placebo yogurt for 12 wk (PPS analysis)^1^^,^^2^

						Linear mixed model, *P* value^3^			
	Group	0 wk	4 wk	8 wk	12 wk	Interaction	4 wk	8 wk	12 wk
Serum IL-6, pg/mL	Placebo	0.442 ± 0.857	0.449 ± 0.859	0.374 ± 0.611	0.466 ± 0.850	0.045	0.861	0.621	0.113
	OLL2712	0.870 ± 1.518	0.627 ± 0.827*	0.645 ± 0.842	0.468 ± 0.796**				
Serum IL-8, pg/mL	Placebo	6.03 ± 2.43	5.24 ± 2.53	5.53 ± 2.51	7.97 ± 6.19**	0.176	NT	NT	0.250
	OLL2712	6.37 ± 2.10	6.03 ± 2.39	5.84 ± 1.97	7.08 ± 2.44				
Serum MCP-1, pg/mL	Placebo	25.2 ± 9.8	22.4 ± 8.0	23.8 ± 8.7	25.2 ± 11.8	0.523	NT	NT	0.053
	OLL2712	34.2 ± 14.3	34.6 ± 13.3	35.1 ± 11.9	35.5 ± 12.5				
Serum TNF-α, pg/mL	Placebo	10.1 ± 4.1	7.7 ± 4.1	10.7 ± 3.2	9.9 ± 4.1	0.653	NT	NT	0.609
	OLL2712	11.4 ± 3.8	9.3 ± 3.7	12.6 ± 4.0	11.2 ± 3.7				
Serum hs-CRP, mg/dL	Placebo	0.066 ± 0.052	0.077 ± 0.054	0.083 ± 0.075	0.086 ± 0.076*	0.972	NT	NT	0.913
	OLL2712	0.071 ± 0.074	0.074 ± 0.062	0.071 ± 0.075	0.088 ± 0.095				
Serum adiponectin, mg/mL	Placebo	4.59 ± 1.81	5.11 ± 2.08	4.85 ± 1.93	4.61 ± 1.74	0.171	NT	NT	0.303
	OLL2712	4.37 ± 163	4.78 ± 1.85	4.47 ± 1.94	4.61 ± 2.03*				

1 Values are means ± SD, *n* = 46 in each group.

2 Significant differences in measurements compared with baseline (0 wk) were determined using the Wilcoxon signed-rank tests (*P < 0.05, **P < 0.01).

3 The group-by-time interaction and intergroup differences were evaluated using a linear mixed model with the model using the amount of change (not including 0 wk) as the response variable, group, time, and group-by-time interaction as fixed effects, the baseline value as the covariate, and time point as a repeated effect.

hs-CRP, high-sensitivity C-reactive protein; MCP-1, monocyte chemotactic protein-1; NT, not tested; PPS, per-protocol set.

### Safety

No serious adverse events were reported in the study, although a total of 29 nonserious adverse events were documented in the participants’ diaries, medical interviews, and blood tests. There were no significant differences in the incidence of adverse events between the groups, and none of these were judged to be associated with the consumption of the test yogurt by the lead physician. The physician determined that the changes in all parameters of the blood and urine tests were within normal limits.

## Discussion

In the present study, we examined whether anti-inflammatory LAB, OLL2712 cells, suppressed chronic inflammation and reduced body fat accumulation in overweight but otherwise healthy individuals. We demonstrated that the ingestion of a test yogurt containing heat-treated OLL2712 cells over 12 wk significantly reduced abdominal fat areas, FPG, and serum IL-6 compared with the placebo yogurt. In addition, we observed a significant increase in body weight and BMI in the placebo group but not in the OLL2712 group.

Abdominal fat areas, as a primary outcome, were assessed using CT, which is considered the gold standard methodology when evaluating body fat. The measurement of body fat percentage using bioelectrical impedance analysis (BIA) is inaccurate and easily fluctuates owing to various uncontrollable confounding factors (33–35). That is, the values of body fat percentage are considered reference values. On the other hand, the measurement of abdominal total fat area using CT is highly accurate and reflects the total body fat mass (36, 37). Therefore, in this study, we set the abdominal total fat area as the primary outcome and evaluated body fat accumulation based on the results obtained by CT analysis. The ingestion of yogurt containing OLL2712 cells for 12 wk reduced the total abdominal fat area by ∼8.5 cm^2^ compared with the placebo yogurt (Table 3**, Supplemental Figure 1**A). The effect size was similar to or slightly lower than that of previous studies (11, 12); however, the participants in these previous studies had larger average visceral fat areas at baseline (127–133 cm^2^ in the active group) than in this study (102 cm^2^ in the OLL2712 group). In the subgroup analysis of the participants with a visceral fat area of 100 cm^2^ or more at screening, the difference between groups in terms of the change in total abdominal fat area over 12 wk was more marked as analyzed by the linear mixed model (difference: 19.0 cm^2^; 95% CI: 8.2, 29.7 cm^2^; *P* = 0.001) (**Supplemental Table 1**). These results demonstrate that the ingestion of OLL2712 cells more prominently reduced the body fat accumulation of participants who were more prone to obesity.

Of the secondary outcomes, changes in FPG significantly differed between groups, and OLL2712 ingestion lowered FPG compared with the placebo (Table 4, **Supplemental Figure 2**A). We acknowledge that baseline FPG concentrations were not uniform in each group because some participants in the placebo group had extremely low FPG at that time. Therefore, we performed a subgroup analysis that excluded participants with baseline FPG below 80 mg/dL; baseline FPG was consistent between subgroups: 86.9 ± 6.1 mg/dL (placebo, *n* = 32) compared with 86.7 ± 5.6 mg/dL (OLL2712, *n* = 43). The 12-wk changes in FPG were significantly less in the OLL2712 group than the placebo group (**Supplemental Figure 3**). HbA1c did not significantly decrease in either group (Table 4), although we recently reported that the ingestion of yogurt containing heat-treated OLL2712 cells for 12 wk significantly reduced HbA1c compared with baseline and the placebo yogurt for prediabetic participants (30). This may be explained by a baseline mean HbA1c of 5.48% in the OLL2712 group in this study, which is within the normal range and considerably lower than in the above-mentioned study (5.86% in the OLL2712 group), such that a significant reduction in HbA1c was unlikely to be detected. However, these results suggest that OLL2712 cells are effective for improving glucose metabolism and can lower FPG.

The reduction in abdominal fat accumulation and FPG by OLL2712 ingestion could be explained by the following physiological mechanisms. We previously demonstrated in animal studies that OLL2712 cells induce IL-10 in intestinal dendritic cells (18) and suppress proinflammatory cytokines in visceral adipose tissue (17, 19). IL-10 is known to suppress proinflammatory cytokines and has been reported to be profoundly implicated in the suppression of chronic inflammation and improvements in glucose and lipid metabolism (20–23). In addition, it was shown that the suppression of proinflammatory cytokines in the intestinal tract leads to the suppression of chronic inflammation in visceral adipose tissue and an improvement in IR (38). These effects suggest that OLL2712 cells induce IL-10 in the intestinal tract to suppress chronic inflammation in visceral adipose tissue, resulting in the improvement of IR. This improvement of IR not only enhances the metabolism of glucose but can also suppress the synthesis of excessive body fat, thereby suppressing its accumulation (4, 5). The validity of this conjecture will be further discussed below.

In the present study, we demonstrated that OLL2712 yogurt reduced serum IL-6 compared with baseline and the placebo yogurt (Table 6, Supplemental Figure 2C). To our knowledge, no previous clinical studies have demonstrated the suppressive effect of serum IL-6 by LAB ingestion in overweight individuals in accordance with the suppression of body fat accumulation. IL-6 is secreted from the adipose tissue in large amounts and is considered a marker of chronic inflammation in the adipose tissue (5). In contrast, serum adiponectin, an anti-inflammatory factor secreted from the adipose tissue in large amounts, significantly increased from 0 to 12 wk only in the OLL2712 group (Table 6). These results suggest that OLL2712 ingestion might suppress chronic inflammation emanating from adipose tissue. Excessive IL-6 production in the adipose tissue is known to be suppressed by inducing IL-10 (5); however, serum IL-10 concentrations were below the detection limit in this study. IL-10 is produced to suppress inflammation, and its biosynthesis is quickly downregulated as inflammation is sufficiently suppressed (22, 39). In addition, serum hs-CRP and IL-8 significantly increased from 0 to 12 wk only in the placebo group (Table 6), which aligns with the results of our previous clinical study (30). CRP is another inflammatory mediator produced by both adipocytes and the liver in response to IL-6 (5). The circulating concentrations of hs-CRP are considered to be a marker for cardiovascular disease risk and have been correlated with IR (5). hs-CRP is also positively associated with obesity, whereas weight loss significantly reduces circulating hs-CRP (40) and is accompanied by improvements in IR (41, 42). Serum IL-8 is also a marker for systemic chronic inflammation (5). Taken together, OLL2712 ingestion may suppress IL-6 expression in the adipose tissue by inducing IL-10 in the intestine, and thereby not exacerbate systemic chronic inflammation.

We suggested that HOMA-IR, a popular IR index, tended to improve in the OLL2712 group in this study (*P* = 0.087 at 8 wk) (Supplemental Figure 2B), which is in agreement with our previous clinical study findings (30). We previously reported that the administration of heat-treated OLL2712 cells significantly reduced blood glucose concentrations after insulin treatment in a mouse model of diet-induced obesity (43). We also reported that the 12-wk ingestion of OLL2712 cells significantly improved HOMA-IR in prediabetic individuals compared with baseline, and the effect was more pronounced in those with chronic inflammation at baseline (24). The ability of OLL2712 ingestion to improve IR was most likely due to the suppression of chronic inflammation.

Conventional yogurt, fermented by cultured *Lactobacillus delbrueckii* subsp. *bulgaricus* and *Streptococcus thermophilus*, is beneficial for human health (44). Prospective studies have shown that yogurt intake is negatively associated with a risk of developing obesity, metabolic syndrome, and T2DM (45–47). Randomized controlled trials in healthy individuals have shown that yogurt intake reduces body fat, serum lipids, FPG, and chronic inflammation compared with a yogurt-free diet (48, 49). It was reported that yogurt intake improved HOMA-IR compared with milk intake and compared with baseline in obese women (26). They speculated that the higher content of lactic acid, conjugated linoleic acid, and folate in yogurt compared with milk might contribute to the beneficial effects (26). In addition, *L. delbrueckii* subsp. *bulgaricus* and *S. thermophilus* included in the yogurt starter culture maintain gut barrier function by regulating the gut microbiome and its metabolites (50, 51). By repairing damaged mucus layers and maintaining tight junctions in the gut, they may contribute to reducing chronic inflammation and lowering the risk of developing obesity (52, 53).

On the other hand, in the present study, the weight and FPG of participants in the placebo yogurt group increased significantly compared with baseline, but not in the OLL2712 group (Tables 3 and 4). The intervention period was from August to December. It has been reported in Japan that the period from summer to winter is generally when people tend to gain weight and when blood glucose and HOMA-IR are more likely to increase (54–57). We confirmed that there were no significant changes in energy intake or physical activity throughout the test period (Table 2). These data suggest that these potential confounding factors beyond yogurt intake have a low impact in this trial and the weight of the participants in the placebo group likely increased due to seasonal variation, which was otherwise suppressed by the intake of the yogurt containing OLL2712 cells. Taken together, we contend that the intake of OLL2712 cells suppressed the accumulation of body fat and attenuated the deterioration of glucose metabolism by suppressing the generation of chronic inflammation associated with an unhealthy lifestyle.

The strength and novelty of this study are as follows: first, the test food in this study was a yogurt formulation, which can be easily ingested daily for a long time. Second, it was demonstrated that the ingestion of heat-treated LAB with anti-inflammatory properties, not limited to *A. muciniphila*, reduced the accumulation of body fat. Third, improvements of the IR index and chronic inflammation markers were also observed in the same trial, suggesting that certain LAB may reduce body fat accumulation by regulating chronic inflammation.

Our trial had some limitations. First, no significant differences were observed in visceral fat area between groups, potentially owing to the relatively small number of participants, and because many participants had low levels of visceral fat at baseline. The ability of OLL2712 to reduce visceral fat could be further clarified by conducting a study with a larger number of participants who are prone to obesity. Second, there was no control group that consumed a test food without yogurt. The placebo yogurt used in this study contained several active ingredients, including >10^11^ cells of *L. delbrueckii* subsp. *bulgaricus* and *S. thermophilus*, which could have provided an antiobesity effect and may have influenced the effect of the OLL2712 cells. Third, no analysis of fecal microbiota or metabolites was performed. The gut microbiota has been associated with the development of obesity, metabolic syndrome, and related metabolic dysfunctions (58). The other limitations were that the increase in type I error rates due to multiple testing could not be denied since many parameters were evaluated as secondary outcomes to support the results of the primary outcome (59), and that we performed a PPS analysis and it might occur that biases associated with not being able to do an ITT analysis, including a decrease in power due to a decrease in the number of cases and not reflecting the influence of some participants. In future studies, we will conduct these additional analyses to clarify the effects and the mechanism of action of OLL2712 cells.

In conclusion, the ingestion of heat-treated OLL2712 cells reduces body fat accumulation and the deterioration of glycemic control and chronic inflammation in overweight but otherwise healthy adults. The results of this study showed that OLL2712 cells might improve glucose and lipid metabolism via the suppression of chronic inflammation.

## Supplementary Material

nzab006_Supplemental_Files

## Notes

Supported by Meiji Co., Ltd. (https://www.meiji.com/global/), which is a food company that manufactures and sells dairy products, confectioneries, and other foods. Meiji Co., Ltd. funded the study and participated in the study design, implementation, interpretation of the data, and writing of this article.

Author disclosures: TT, AG, TS, KF, and YA are employees of Meiji Co., Ltd. SH, NS, and SS conducted the research funded by Meiji Co., Ltd.

Supplemental Table 1 and Supplemental Figures 1–3 are available from the “Supplementary data” link in the online posting of the article and from the same link in the online table of contents at https://academic.oup.com/cdn/.

Abbreviations used: CT, computed tomography; FPG, fasting plasma glucose; HbA1c, glycated hemoglobin; hs-CRP, high-sensitivity C-reactive protein; IR, insulin resistance; ITT, intent-to-treat; LAB, lactic acid bacteria; OLL2712, *Lactobacillus plantarum* OLL2712; PPS, per-protocol set; TLR2, toll-like receptor 2; T2DM, type-2 diabetes mellitus; UMIN, University Hospital Medical Information Network.
