# Supplementary material for: Ingesting Yogurt Containing Lactobacillus plantarum OLL2712 Reduces Abdominal Fat Accumulation and Chronic Inflammation in Overweight Adults in a Randomized Placebo-Controlled Trial
Source: Curr Dev Nutr. 2021 Feb 3;5(2):nzab006. doi: 10.1093/cdn/nzab006 (PMC7937491; doi:10.1093/cdn/nzab006)
Supplement: nzab006_Supplemental_Files [file nzab006_supplemental_files.zip › Supplemental_Figure_1.pptx]

## Slide 1
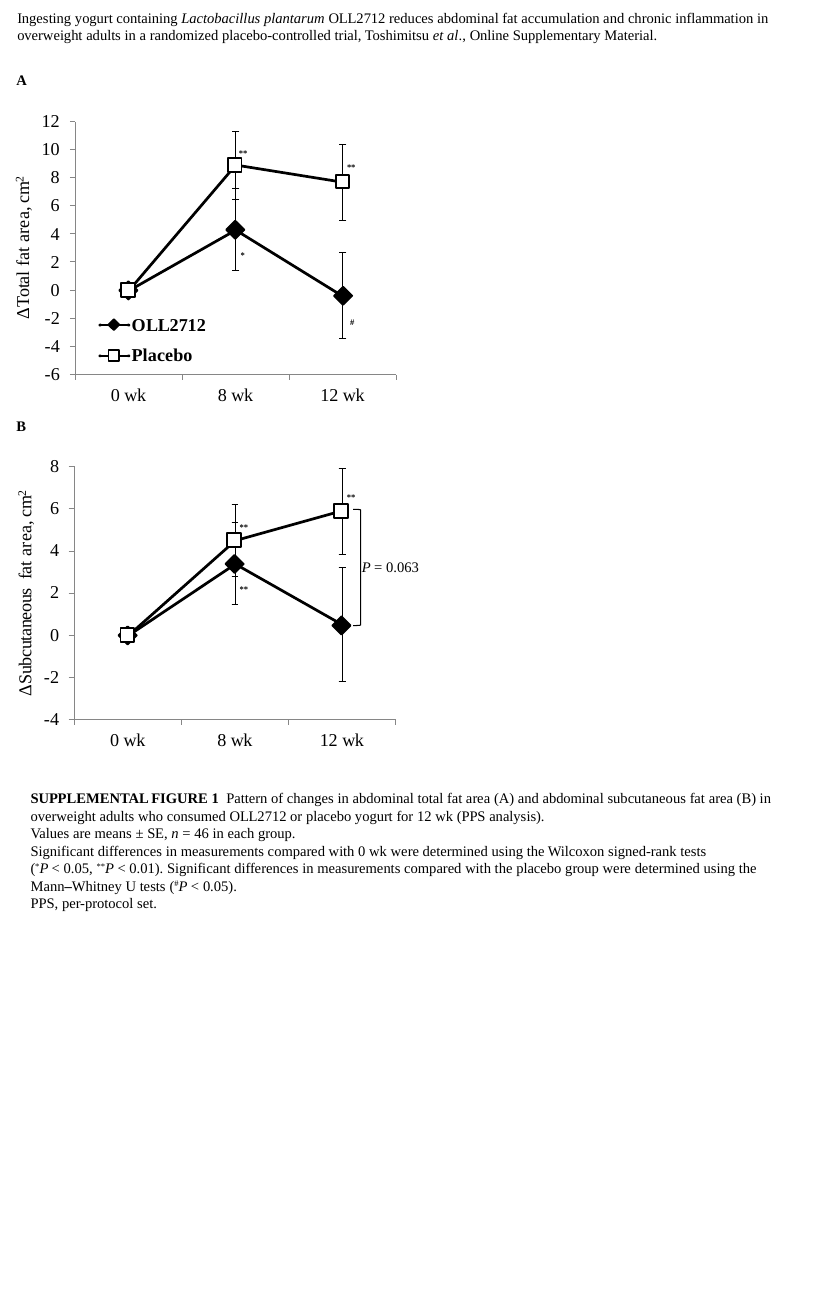

Ingesting yogurt containing Lactobacillus plantarum OLL2712 reduces abdominal fat accumulation and chronic inflammation in overweight adults in a randomized placebo-controlled trial, Toshimitsu et al., Online Supplementary Material.
A
**
**
*
#
B
**
**
P = 0.063
**
SUPPLEMENTAL FIGURE 1 Pattern of changes in abdominal total fat area (A) and abdominal subcutaneous fat area (B) in overweight adults who consumed OLL2712 or placebo yogurt for 12 wk (PPS analysis).
Values are means ± SE, n = 46 in each group.
Significant differences in measurements compared with 0 wk were determined using the Wilcoxon signed-rank tests
(*P < 0.05, **P < 0.01). Significant differences in measurements compared with the placebo group were determined using the Mann–Whitney U tests (#P < 0.05).
PPS, per-protocol set.
