# Supplementary material for: Ingesting Yogurt Containing Lactobacillus plantarum OLL2712 Reduces Abdominal Fat Accumulation and Chronic Inflammation in Overweight Adults in a Randomized Placebo-Controlled Trial
Source: Curr Dev Nutr. 2021 Feb 3;5(2):nzab006. doi: 10.1093/cdn/nzab006 (PMC7937491; doi:10.1093/cdn/nzab006)
Supplement: nzab006_Supplemental_Files [file nzab006_supplemental_files.zip › Supplemental_Figure_2.pptx]

## Slide 1
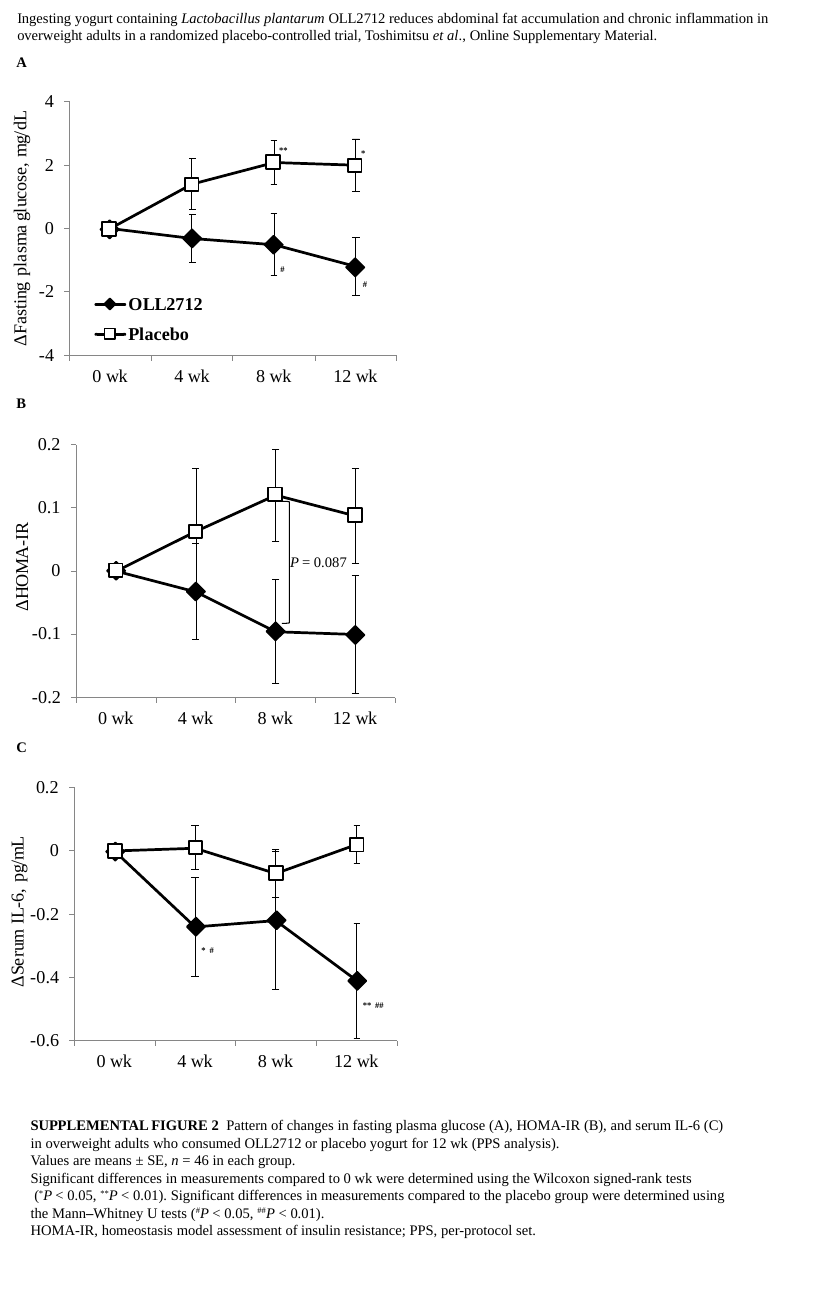

Ingesting yogurt containing Lactobacillus plantarum OLL2712 reduces abdominal fat accumulation and chronic inflammation in overweight adults in a randomized placebo-controlled trial, Toshimitsu et al., Online Supplementary Material.
A
**
*
#
#
B
P = 0.087
C
* #
** ##
SUPPLEMENTAL FIGURE 2 Pattern of changes in fasting plasma glucose (A), HOMA-IR (B), and serum IL-6 (C)
in overweight adults who consumed OLL2712 or placebo yogurt for 12 wk (PPS analysis).
Values are means ± SE, n = 46 in each group.
Significant differences in measurements compared to 0 wk were determined using the Wilcoxon signed-rank tests
 (*P < 0.05, **P < 0.01). Significant differences in measurements compared to the placebo group were determined using the Mann–Whitney U tests (#P < 0.05, ##P < 0.01).
HOMA-IR, homeostasis model assessment of insulin resistance; PPS, per-protocol set.
