# Supplementary material for: Ingesting Yogurt Containing Lactobacillus plantarum OLL2712 Reduces Abdominal Fat Accumulation and Chronic Inflammation in Overweight Adults in a Randomized Placebo-Controlled Trial
Source: Curr Dev Nutr. 2021 Feb 3;5(2):nzab006. doi: 10.1093/cdn/nzab006 (PMC7937491; doi:10.1093/cdn/nzab006)
Supplement: nzab006_Supplemental_Files [file nzab006_supplemental_files.zip › Supplemental_Figure_3.pptx]

## Slide 1
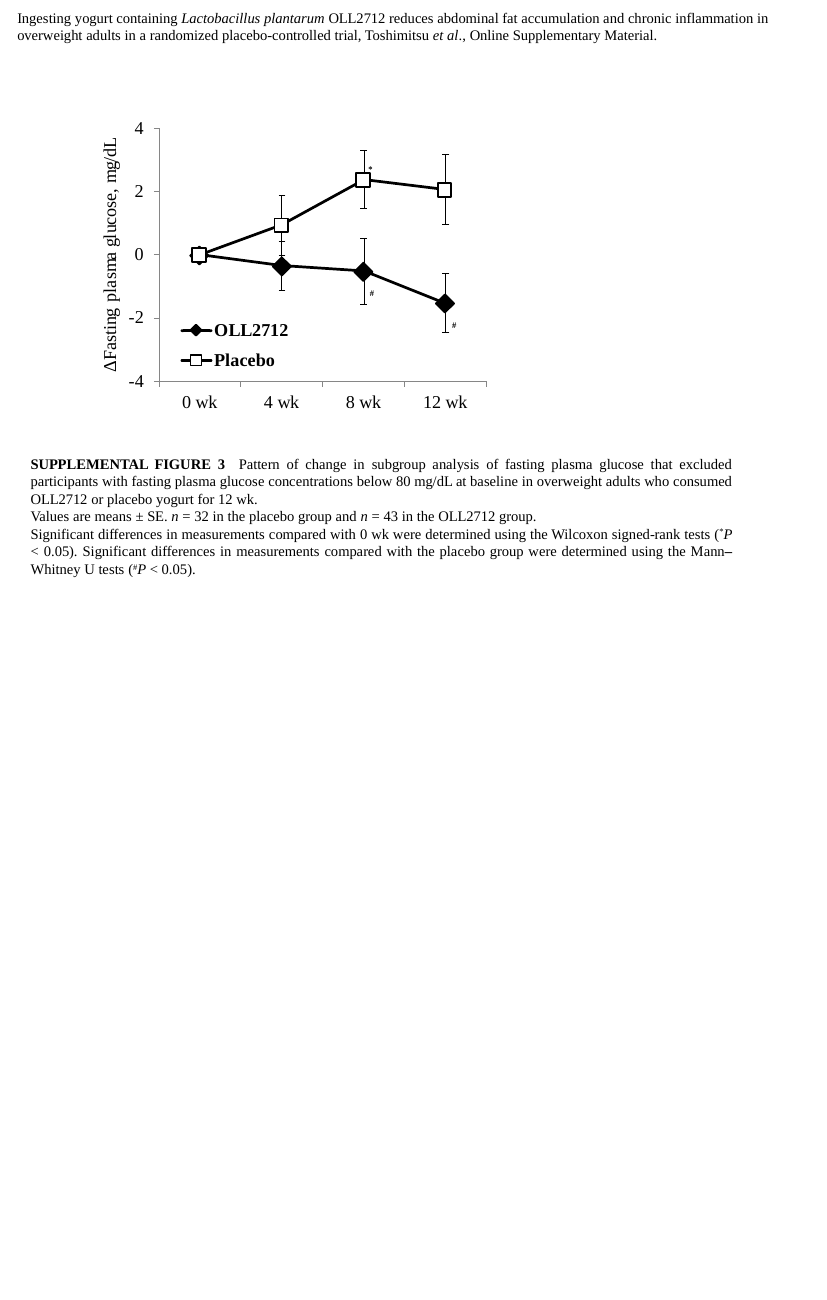

Ingesting yogurt containing Lactobacillus plantarum OLL2712 reduces abdominal fat accumulation and chronic inflammation in overweight adults in a randomized placebo-controlled trial, Toshimitsu et al., Online Supplementary Material.
*
#
#
SUPPLEMENTAL FIGURE 3 Pattern of change in subgroup analysis of fasting plasma glucose that excluded participants with fasting plasma glucose concentrations below 80 mg/dL at baseline in overweight adults who consumed OLL2712 or placebo yogurt for 12 wk.
Values are means ± SE. n = 32 in the placebo group and n = 43 in the OLL2712 group.
Significant differences in measurements compared with 0 wk were determined using the Wilcoxon signed-rank tests (*P < 0.05). Significant differences in measurements compared with the placebo group were determined using the Mann–Whitney U tests (#P < 0.05).
