# Supplementary material for: Ingesting Yogurt Containing Lactobacillus plantarum OLL2712 Reduces Abdominal Fat Accumulation and Chronic Inflammation in Overweight Adults in a Randomized Placebo-Controlled Trial
Source: Curr Dev Nutr. 2021 Feb 3;5(2):nzab006. doi: 10.1093/cdn/nzab006 (PMC7937491; doi:10.1093/cdn/nzab006)
Supplement: nzab006_Supplemental_Files [file nzab006_supplemental_files.zip › Supplemental_Table_1.docx]

**SUPPLEMENTAL TABLE 1** Subgroup analysis of body fat and anthropometrics measurements in participants with an abdominal visceral fat area of 100 cm^2^ or more at screening who consumed OLL2712 or placebo yogurt for 12 wk^1,2^

|  | **Group** | **0 wk** | **4 wk** | **8 wk** | **12 wk** | **Linear mixed model, p-value ^3^** | | | |
| --- | --- | --- | --- | --- | --- | --- | --- | --- | --- |
|  |  |  |  |  |  | **Interaction** | **4 wk** | **8 wk** | **12 wk** |
| Abdominal total fat area, cm^2^ | Placebo | 344.4 ± 61.1 | NA | 352.1 ± 60.7 | 354.3 ± 59.1^**^ | 0.069 | NA | NT | 0.001 |
|  | OLL2712 | 312.5 ± 62.1 | NA | 313.5 ± 60.1 | 307.0 ± 56.1 |  |  |  |  |
| Abdominal visceral fat area, cm^2^ | Placebo | 121.8 ± 17.0 | NA | 124.7 ± 19.0 | 124.1 ± 18.6 | 0.610 | NA | NT | 0.127 |
|  | OLL2712 | 111.4 ± 14.7 | NA | 112.0 ± 19.1 | 109.5 ± 17.6 |  |  |  |  |
| Abdominal subcutaneous fat area, cm^2^ | Placebo | 222.5 ± 53.3 | NA | 227.4 ± 54.3^*^ | 230.2 ± 53.8^**^ | 0.006 | NA | 0.097 | 0.005 |
|  | OLL2712 | 201.1 ± 59.6 | NA | 201.4 ± 58.0 | 197.5 ± 53.6 |  |  |  |  |
| Weight, kg | Placebo | 78.4 ± 8.8 | 78.9 ± 8.9 | 79.1 ± 9.0 | 78.8 ± 8.5 | 0.838 | NT | NT | 0.329 |
|  | OLL2712 | 73.6 ± 7.3 | 73.7 ± 7.4 | 73.7 ± 7.2 | 73.5 ± 7.1 |  |  |  |  |
| BMI, kg/m^2^ | Placebo | 27.8 ± 1.5 | 27.9 ± 1.5 | 28.0 ± 1.6 | 27.9 ± 1.6 | 0.913 | NT | NT | 0.225 |
|  | OLL2712 | 27.2 ± 1.4 | 27.2 ± 1.4 | 27.3 ± 1.4 | 27.2 ± 1.4 |  |  |  |  |
| Body fat, % | Placebo | 31.9 ± 5.9 | 32.1 ± 6.1 | 33.1 ± 5.9 | 33.5 ± 6.1^**^ | 0.234 | NT | NT | 0.228 |
|  | OLL2712 | 30.9 ± 5.7 | 31.0 ± 5.8 | 31.3 ± 6.0 | 31.9 ± 5.7^*^ |  |  |  |  |
| Waist circumference, cm | Placebo | 95.1 ± 5.0 | 95.0 ± 4.8 | 94.7 ± 5.2 | 94.6 ± 4.7 | 0.235 | NT | NT | 0.230 |
|  | OLL2712 | 92.9 ± 4.3 | 92.6 ± 4.9 | 92.5 ± 4.6 | 91.7 ± 4.8^**^ |  |  |  |  |
| Hip circumference, cm | Placebo | 100.5 ± 4.0 | 100.4 ± 3.7 | 100.2 ± 3.8 | 100.3 ± 4.0 | 0.212 | NT | NT | 0.598 |
|  | OLL2712 | 98.3 ± 3.6 | 98.5 ± 4.1 | 98.1 ± 3.9 | 97.9 ± 3.8 |  |  |  |  |
| Waist-to-hip ratio | Placebo | 0.946 ± 0.045 | 0.947 ± 0.036 | 0.945 ± 0.039 | 0.944 ± 0.036 | 0.203 | NT | NT | 0.251 |
|  | OLL2712 | 0.945 ± 0.034 | 0.941 ± 0.037 | 0.944 ± 0.033 | 0.937 ± 0.036^*^ |  |  |  |  |

^1^Values are means ± SD, *n* = 24 in the placebo group and *n* = 23 in the OLL2712 group.

^2^Significant differences in measurements compared to baseline (0 wk) were determined using the paired samples t-tests or the Wilcoxon signed-rank tests (^*^*P* < 0.05, ^**^*P* < 0.01).

^3^The group-by-time interaction and inter-group differences at each time point were evaluated using a linear mixed model with the model using the amount of change (not including 0 wk) as the response variable, group, time, and group-by-time interaction as fixed effects, the baseline value as covariate, and time point as a repeated effect.

BMI, body mass index; NA, not assessed; NT, not tested.
